# Efficacy and safety of the Miethke programmable differential pressure valve (*proGAV®2.0*): a single-centre retrospective analysis

**DOI:** 10.1007/s00381-021-05162-3

**Published:** 2021-05-21

**Authors:** Benjamin J. Hall, Conor S. Gillespie, Dawn Hennigan, Veejay Bagga, Conor Mallucci, Benedetta Pettorini

**Affiliations:** 1 Aintree University Hospital, Liverpool University Hospitals NHS Foundation Trust, Liverpool, L9 7AL UK; 2 The University of Liverpool, Liverpool, L69 3BX UK; 3 Alder Hey Children’s Hospital, NHS Foundation Trust, Liverpool, L14 5AB UK

**Keywords:** Hydrocephalus, Over-drainage, ProGAV®2.0, Valve

## Abstract

**Purpose:**

Achieving decompression without CSF over-drainage remains a challenge in hydrocephalus. Differential pressure valves are a popular treatment modality, with evidence suggesting that incorporation of gravitational units helps minimise over-drainage. This study seeks to describe the utility of the *proGAV®2.0* programmable valve in a paediatric population.

**Methods:**

Clinical records and imaging of all patients fitted with *proGAV®2.0* valves and Miethke fixed-pressure valves between 2014 and 2019 at our tertiary centre were analysed. Patient demographics, indication for shunt and valve insertion/revision and time to shunt/valve revision were collected. Ventricular linear metrics (fronto-occipital horn ratio (FOHR) and fronto-occipital horn width ratio (FOHWR)) were collected pre- and post-valve insertion. Microsoft Excel and SPSS v24 were used for data collection and statistical analysis.

**Results:**

Eighty-eight *proGAV®2.0* valves were inserted in a population of 77 patients (*n* = 45 males (58%), mean age 5.1 years (IQR: 0.4–11.0 years)). A total of 102 Miethke fixed-pressure valves were inserted over the same time period. Median follow-up was 17.5 months (1.0–47.3). One (1.1%) *proGAV®2.0* was revised due to over-drainage, compared to 2 (1.9%) fixed-pressure valves (*p* > 0.05). *ProGAV®2.0* insertion resulted in a significant decrease in the mean number of revisions per patient per year (1.77 vs 0.25; *p* = 0.01). Overall shunt system survival with the *proGAV®2.0* was 80.4% at 12 months, and mean time to revision was 37.1 months, compared to 31.0 months (95%CI: 25.7–36.3) and 58.3% in fixed-pressure valves (*p* < 0.01). Significant decreases were seen following *proGAV®2.0* insertion in both FOHR and FOHWR, by 0.014 (95%CI: 0.006–0.023, *p* = 0.002) and 0.037 (95%CI: 0.005–0.069, *p* = 0.024) respectively.

**Conclusion:**

The *proGAV®2.0* provides effective decompression of hydrocephalic patients, significantly reduces the number of valve revisions per patient and had a significantly greater mean time to revision than fixed-pressure valves.

## Introduction

Ventricular shunts have long formed the mainstay of hydrocephalus treatment with ventriculo-peritoneal shunts (VPS) proving the most popular, utilised in an estimated 3500 procedures annually in the UK [1]. Ventricular shunting is generally accepted to be associated with more complications than any other neurosurgical intervention, including valve or catheter blockage, breakage, infection [1, 2] and increasingly reported over-drainage [3–6]. The combined effects of hydrostatic forces within CSF and gravity in the upright position result in a siphon effect on CSF, leading to excess drainage of fluid from the ventricles [7, 8]. In the developing brain, such over-drainage has been demonstrated to affect cerebral morphology, leading to premature synostosis and microcephaly at the detriment of normal neurodevelopment [4, 5]. In order to mitigate this phenomenon and better mimic physiological CSF drainage, the use of differential pressure valves has become a standard practice when fitting VPS [8].

The Miethke *proGAV®2.0* comprises an adjustable differential pressure unit (DPU) composed of a solid titanium body with a ball-in-cone valve. A pre-tensioned bow spring, externally adjustable via magnets, defines the opening pressure of the DPU. The pre-tensioning of the spring and thus the opening pressure can be adjusted by turning a rotor, with the valve implanted under the patient’s skin. Distal to the DPU lies a gravitational unit of fixed resistance, governed by the weight of a tantalum ball. When the patient is lying down, the gravitational unit remains open, and therefore CSF outflow from the skull meets little resistance. Hence, the opening pressure of the *proGAV®2.0* is defined by the setting of the adjustable DPU. When the patient moves into an upright position, the gravitational unit closes. In addition to the opening pressure of the adjustable DPU, the weight of the tantalum ball has to be exceeded; thus, the opening pressure becomes the sum of both DPU and gravitational unit. Only when the sum of the intraventricular pressure and the hydrostatic pressure exceeds the opening pressure of the combined components is drainage permitted [9].

Typically, failure of a fixed-pressure valve system requires valve replacement which is accompanied by the risk of complication. Limiting the need for repeat surgical intervention is a crucial advantage to programmable valves; however, they are accompanied by their own complications [6, 10, 11], and there is evidence to suggest that shunt survival is not significantly different from that of fixed-pressure valves [12, 13]. Given their complex mechanisms, these valves are typically more expensive than their fixed-pressure counterparts, and the debate over the cost-benefit is ongoing [10].

In complex patients for whom multiple valve revisions are required, the need to minimise the number of operations is crucial, and recent data from our centre has demonstrated the positive impact of using fixed-pressure valves in treating hydrocephalus [14]. This study aims to describe the experience of a tertiary paediatric neurosurgical centre in using the *proGAV®2.0* programmable valve.

## Methods

Clinical notes of all patients receiving *proGAV®2.0* valves at either initial insertion or revision of shunts at Alder Hey Children’s Hospital NHS Foundation Trust between January 2014 and September 2018 were collected. Data was collected according to the following headings: (i) patient demographics, (ii) symptomatology (pre and postoperatively), (iii) indication for shunt/valve insertion, (iv) frequency of surgical intervention, (v) frequency of valve setting alteration and (vi) ventricular linear metrics.

For comparison, Miethke fixed differential pressure valve survival data for those inserted between January 2014 and September 2018 was extracted from our centre’s database [15]. Miethke paediGAV, GAV and DualSwitch valves were assessed. Patient demographics, ventricular metrics and shunt revision rate pre- and post-insertion were not available for fixed-pressure valves.

Shunt failure was defined as a surgical intervention wherein a valve or entire shunt system was replaced. Systems in situ for <1 month were excluded from survival analysis to account for immediate postoperative or inpatient-related complications. Kaplan-Meier curves were produced for (i) overall shunt system survival, (ii) shunt system survival (excluding infection and distal failure), (iii) de novo vs replacement valve survival and (iv) ≤12 months vs >12 months on valve insertion.

Over-drainage was defined according to a combination of ventricular morphology, symptomatic presentation of the patient and, if performed, ICP monitor values. The rationale for selecting appropriate replacement valves in the cases where revision was necessary was decided on a case-by-case basis. Gravitational unit choice was made according to Miethke guidance: 20cmH2O for those under 5 years of age and 25cmH2O for over 5 years. DPU pressure setting on insertion was decided on a case-by-case basis, guided by Miethke manufacturer recommendations ranging from 5cmH2O to 10cmH2O and above in those with particularly wide ventricles. Adjustments to the DPU pressure setting were made according to either inadequate response in ventricular metrics, the patient remaining symptomatic or ICP monitor values.

For all patients, preoperative and postoperative imaging was assessed to determine changes in ventricular linear metrics. T2-weighted MRI scans were used to measure frontal and occipital horn ratio (FOHR) and frontal and occipital horn width ratio (FOHWR). In the absence of T2-weighted MRI, CT scans were used. All measurements were taken at the level of the intraventricular foramen of Monro, as identified on axial images. The FOHR was defined as the mean value of the frontal and occipital horn width divided by twice the widest biparietal diameter (BPD) [16, 17]. FOHWR was then defined as the average of the maximum width of the individual frontal and occipital horns, divided by twice the widest BPD [15]. The closest available scans prior to and following shunt insertion/revision were used to assess any changes.

The latest versions of Microsoft Excel and SPSS statistical software were used for data collection and statistical analysis respectively. Kaplan-Meier curves and log-rank tests were used to assess survival outcomes. Chi-squared test was used to compare categorical variables. A threshold of *p* = 0.05 was used to determine significance.

## Results

### Patient population

Seventy-seven patients were identified as having received a *proGAV®2.0* valve, of which *n* = 45 (58%) were male. The overall mean age of the cohort was 5.1 years (IQR: 0.4–11.0 years). Median age for de novo *proGAV®2.0* insertion was 11 months and for revision was 60 months. The valve population was dichotomised into groups ≤12 months of age (*n* = 35, 39.8%) and >12 months of age (*n* = 53, 60.2%).

### Valve data

Median follow-up was 17.5 months (1.0–47.3 months). Eighty-eight *proGAV®2.0* valves were inserted over the time period analysed; therefore, *n* = 88 cases were available for assessment. Of these, *n* = 42 (48%) were as part of de novo shunt insertions and the remainder as revisions (*n* = 46, 52%). Sixty-six (86%) patients received *n* = 1 *proGAV® 2.0*, and 11 (14%) patients received *n* = 2. Causes of hydrocephalus that indicated initial shunt insertion included intraventricular haemorrhage (*n* = 22, 28.6%), spina bifida (*n* = 8, 10.4%), tumours (*n* = 5, 6.5%) and aqueductal stenosis (*n* = 4, 5.2%) (Table 1).

**Table 1:** The indications for insertion of a proGAV2.0 valve as part of a de novo shunt system

Indication for **initial shunt insertion**	**Frequency (*n*; %)**
IVH	**22** (28.6)
Syndromic/craniofacial	**7** (9.1)
Spina bifida/myelomeningocoele	**8** (10.4)
Post traumatic/vascular	**3** (3.9)
Tumour	**5** (6.5)
Chiari/DWS	**6** (7.8)
Aqueductal stenosis	**4** (5.2)
Congenital NOS	**11** (14.3)
Infection	**3** (3.9)
IIH	**2** (2.6)
Unknown	**6** (7.8)

Indications for replacement of other valves with *proGAV®2.0*s included valve obstruction (*n* = 12, 35%), infection (*n* = 4, 12%) and over-drainage (*n* = 9, 26%) (Table 2). No patients who received a *proGAV®2.0* for over-drainage had recurrence of their symptoms after insertion during follow-up.

**Table 2:** The indications for revision of different valve subtypes in our cohort

Indication for **shunt revision**	proGAV 2.0 (*n* = 20 (%))	Other valves in this series (*n* = 34 (%))	Miethke non-programmable (*n* = 42 (%))	Aetiology as a proportion of all shunt failures (%)
Ventricular catheter blockage	**2** (10)	**7** (21)	**15** (36)	**25**
Valve obstruction/ underdrainage	**11** (55)	**12** (35)	**14** (33)	**39**
Valve mechanism failure	**3** (15)	**1** (3)	n/a	**4**
Over-drainage	**0**	**9** (26)	**2** (5)	**11**
Infection	**2** (10)	**4** (12)	**10** (24)	**17**
Wound breakdown	**1** (5)	**0**	**0**	**1**
Other/NOS	**1** (5)	**1** (3)	**1** (2)	**3**

*N* = 20 (23%) *proGAV®2.0* valves required revision. Obstruction was the leading indication for *proGAV®2.0* revision (*n* = 11, 55%), followed by valve mechanism failure (*n* = 3, 15%) and catheter failure (*n* = 2, 10%) (Table 2). *ProGAV®2.0s* were exchanged ‘like for like’ in *n* = 9 (20%) cases. Of these, indications for revision were valve blockage (*n* = 6, 67%), catheter/distal tubing problem (*n* = 2, 22%) and infection (*n* = 1, 11%). *N* = 5 (56%) of *proGAV®2.0* ‘like for like’ revisions remained in situ at the latest data collection.

A total of 102 patients received a variety of Miethke fixed-pressure valves during the same time period: 4/24 (*n* = 14, 13.7%), 9/24 (*n* = 2, 19.6%), 9/29 (*n* = 82, 80.4%), 10/40 (*n* = 3, 2.9%) and DualSwitch (*n* = 1, 1%). Over-drainage led to revision in *n* = 2 (1.9%) fixed-pressure valves. *N* = 42 required revision, due to valve obstruction (*n* = 14, 33%), infection (*n* = 10, 24%) and over-drainage (*n* = 2, 5%) (Table 2).

The only significant difference in aetiology of failure between valve types was over-drainage, occurring significantly more often in the series of valves replaced by a *proGAV®2.0* (*n* = 9, 26%) than in either *proGAV®2.0* (*n* = 1, 1.1%) or Miethke fixed-pressure valves (*n* = 2, 1.9%) (*p* < 0.01).

### Symptomatology

Each *proGAV®2.0* inserted (*n* = 88) was treated as an individual case when analysing symptomatology before and after valve insertion, of which *n* = 78 (89%) had available data (*n* = 33 ≤12 months; *n* = 45 >12 months). In those ≤12 months, increasing head circumference, vomiting, lethargy, behavioural changes and sunsetting occurred most frequently. In those >12 months of age, headache, vomiting, nausea, lethargy and behavioural changes were most common. All patients found to be over-draining presented with low-pressure headaches (Table 3).

**Table 3.** Clinical presentation data of those receiving proGAV2.0 valves, grouped according to age

**Symptom/sign**	**Frequency** (>12 mo) (*n* = 45, %)	**Improvement (*n*, %)**	**No change (*n*, %)**
Headache *	**14** (31)	**12** (86) *	**1** (7)
Nausea/vomiting	**13** (29)	**12** (92)	**1** (8)
Behavioural/memory changes *	**7** (16)	**5** (71) *	**1** (14)
Low-pressure symptoms	**6** (13)	**5** (100)	**1** (17)
Lethargy	**4** (9)	**4** (100)	**0**
GCS drop/collapse	**4** (9)	**4** (100)	**0**
Visual change	**4** (9)	**3** (75)	**1** (25)
Focal neurology/co-ordination	**3** (7)	**3** (100)	**0**
Seizure	**2** (4)	**2** (100)	**0**
Papilloedema	**2** (4)	**2** (100)	**0**
Other	**2** (4)	**2** (100)	**0**
**Symptom/sign**	**Frequency** (≤12 mo) (*n* = 33, %)	**Improvement (*n*, %)**	**No change (*n*, %)**
Increasing head circumference*	**19** (58)	**17** (89) *	**0**
Bulging fontanelle/splayed sutures	**11** (33)	**11** (100)	**0**
Sunsetting	**6** (18)	**6** (100)	**0**
Lethargy	**5** (15)	**5** (100)	**0**
Nausea/vomiting	**3** (9)	**3** (100)	**0**
Subgaleal collection/myelo-meningocoele*	**5** (15)	**3** (60) *	**0**
Behavioural change	**2** (6)	**2** (100)	**0**
Other	**5** (15)	**5** (100)	**0**

‘Other’ includes neck pain (*n* = 1) and systemic signs of infection (*n* = 1) in the >12-month group and systemic signs of infection (*n* = 2), nystagmus (*n* = 1), abdominal failure/swelling (*n* = 1) and episodes of oxygen desaturation (*n* = 1) in the ≤12-month group

*Follow-up data to determine resolution of symptoms was not available for *n* = 5 cases across the entire cohort in several categories

### ICP monitoring

*N* = 11 of 88 (12.5%) had available ICP monitoring data pre-insertion of *proGAV®2.0*, and *n* = 5 (5.7%) had available post-insertion data. Pre-insertion, *n* = 6 (54.5%) had raised ICP, and *n* = 5 (45.5%) had low ICP. Post-insertion, *n* = 1 (20%) was normal, *n* = 1 (20%) had high pressure and *n* = 3 (60%) low pressure (1 of which resulted in further valve revision).

### Pressure settings

*N* = 44 (57.1%) patients were under 5 years and therefore received 20cmH20 gravitational units and *n* = 33 (42.9%) over 5 years and therefore received 25cmH20 gravitational units. Initial pressure setting data for the DPU of newly inserted *proGAV®2.0s* was available for *n* = 69 valves, of which the median was 10cmH2O (range: 5–14). Over the time period analysed, ‘pressure alteration’ data was available for *n* = 71 valves; *n* = 41 (57.7%) of which required no alteration. Of the 30 valves (42.3%) requiring adjustment, *n* = 16 (22.5%) required 1 adjustment, *n* = 8 (11.2%) required 2 adjustments and *n* = 6 patients (8.5%) required ≥3 adjustments.

### Valve survival

**Fig. 1:**
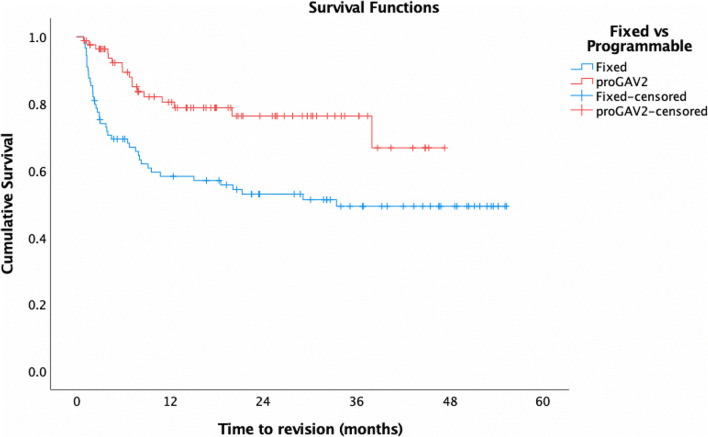
Comparative cumulative survival of the *proGAV®2.0* valve (red) and Miethke fixed-pressure valves (blue)

**Fig. 2:**
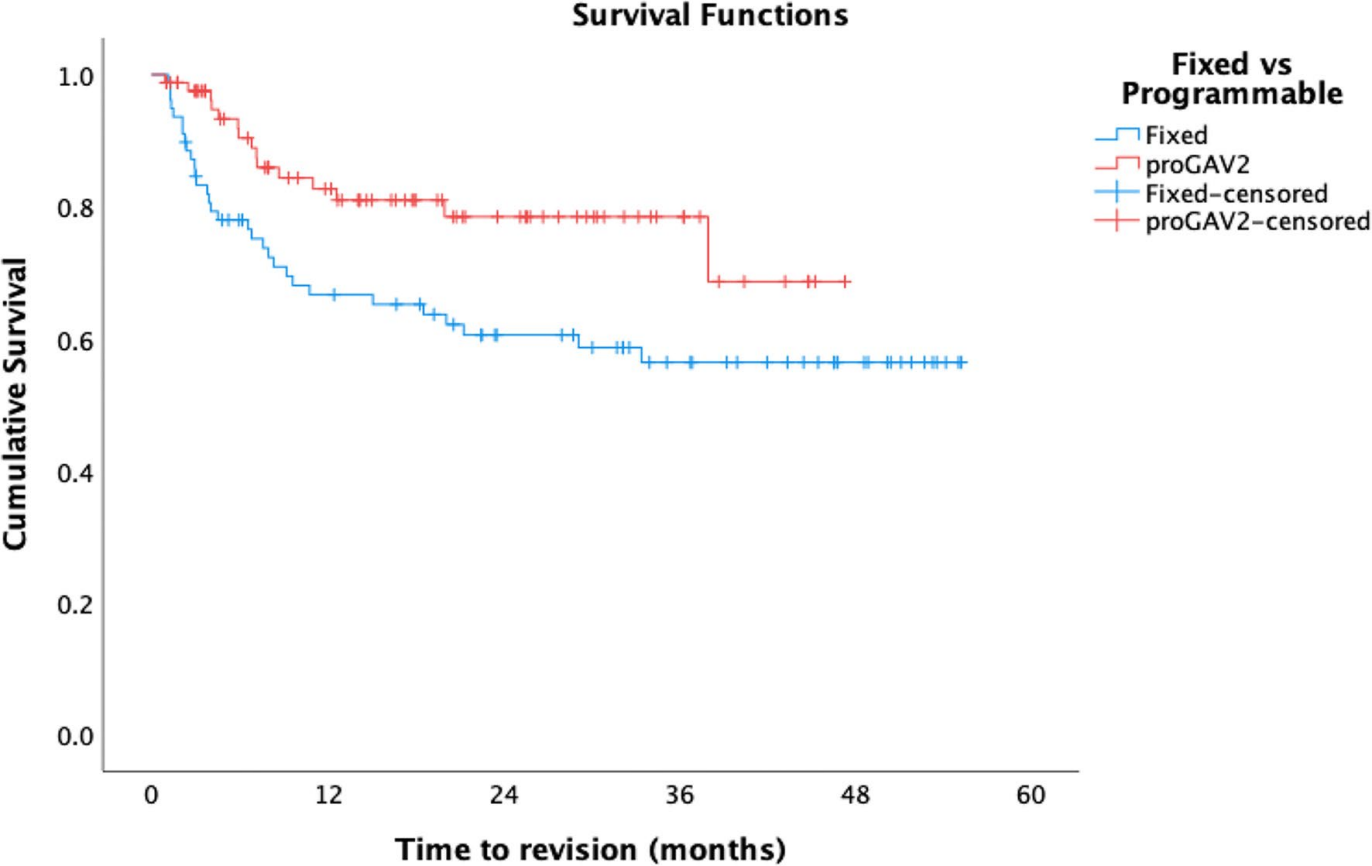
Comparative cumulative survival of the *proGAV®2.0* valve (red) and Miethke fixed-pressure valves (blue) with exclusion of all failures due to infection, distal catheter or cause not otherwise specified

*N* = 3 (3.4%) *proGAV®2.0* and *n* = 13 (12.7%) fixed-pressure valves were replaced within 1 month of insertion and so were excluded from further analysis. Overall mean time to valve revision in the *proGAV®2.0* group was 37.1 months (95%CI: 32.8–41.3), and overall survival (OS) was 80.4% at 12 months compared to 31.0 months (95%CI: 25.7–36.3) and 58.3% at 12 months in the fixed valve group (Fig. 1) (*p* = 0.001). After excluding infection and distal failure as cause of failure, mean time to revision rose to 38.0 months (95%CI: 33.8–42.2) and cumulative survival to 82.7% with a *proGAV®2.0,* compared to 35.1 months (95%CI: 29.6–40.6) and 66.6% at 12 months with fixed-pressure valves (*p* = 0.02) (Fig. 2).

**Fig. 3:**
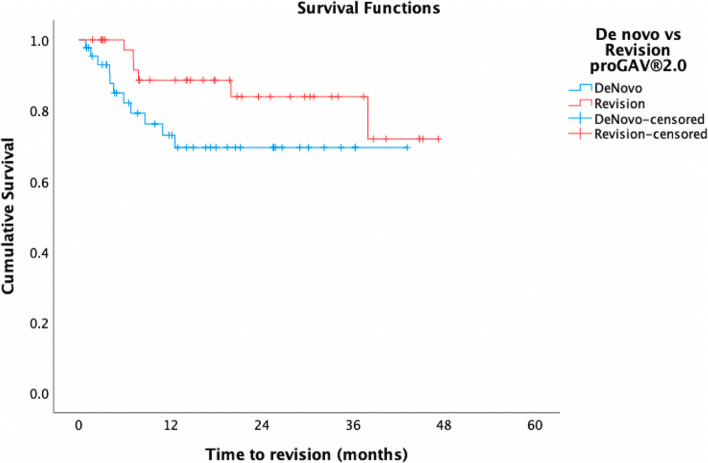
Cumulative survival of de novo (blue) vs revised (red) *proGAV®2.0* valves

**Fig. 4:**
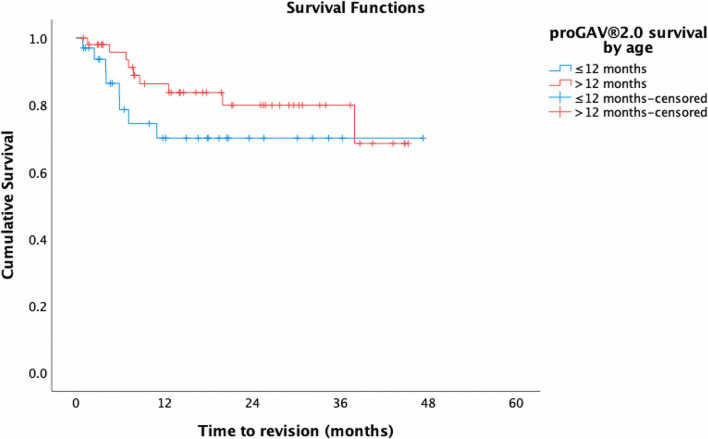
Cumulative survival of *proGAV®2.0* valves in patients ≤12 months of age on insertion (blue) vs those >12 months (red)

Within the *proGAV®2.0* cohort alone, de novo valves had a mean time to revision of 31.9 months and 12-month cumulative survival of 73%, compared to a mean time to revision of 40.3 months and 12-month survival of 88.6% in revised valves (*p* = 0.10) (Fig. 3). Of the *proGAV®2.0* valves exchanged ‘like for like’, mean survival of the first valve was shorter than the second (7.3 vs 11.9 months), though not significantly so (*p* > 0.1). *ProGAV®2.0s* in patients ≤12 months had a mean OS of 35.3 months and cumulative survival at 12 months of 71.2%, compared to a mean OS of 37.3 months and 86.3% 12-month cumulative survival in those >12 months (*p* = 0.25) (Fig. 4).

### Ventricular metrics

Across the entire cohort of *proGAV®2.0* insertions, a statistically significant decrease was seen in both mean FOHR and FOHWR. The mean decrease in FOHR was 0.014 (95%CI: 0.006–0.023, *p* = 0.002), whilst the mean decrease in FOHWR was 0.037 (95%CI: 0.005–0.069, *p* = 0.024) (Table 4).

**Table 4:** Change in ventricular size following insertion of *proGAV®2.0* differential pressure valves as defined by fronto-occipital horn ratio and fronto-occipital horn width ratio

**Ventricular metric**	**Mean change in metric**	**95% CI**	***p*** **value**
**FOHR**	−0.014	0.006–0.023	**0.002**
**FOHWR**	−0.037	0.005–0.069	**0.024**

Across the cohort, *n* = 5 (6.5%) had slit ventricles before and after treatment (all of which were valve revisions). *N* = 4 of these of these had measurable ventricular metrics, of which none changed significantly following insertion; however, all of these patients had improvement in their symptoms.

## Discussion

This study aims to describe a tertiary paediatric neurosurgical centre’s experience with the *proGAV®2.0* programmable valve.

Across the *proGAV®2.0* cohort, a marked improvement in symptoms was observed after valve insertion, accompanied by a significant decrease in ventricular metrics (*p* = 0.002, *p* = 0.024). In patients with slit ventricles and symptoms of over-drainage, despite clinically improving, little real change was seen in their ventricular metrics. Slit-ventricle syndrome is relatively poorly understood, and the persistence of slit ventricles despite intervention and clinical improvement may speak to the nature of ventricular compliance in the syndrome itself or perhaps suggest that FOHR and FOHWR are not useful metrics in this scenario. Regardless, further investigation is needed into slit-ventricle syndrome both independently and in relation to different valve/shunt systems.

Valve revision forms a significant part of the paediatric neurosurgical workload, and numerous sources have reviewed the risks associated with it [18–22]. Theoretically mitigating the need for shunt revision is therefore an attractive feature of programmable valves, and in this cohort the insertion of a *proGAV®2.0* valve resulted in a decrease in mean revision rate from 1.77 to 0.25 per patient per year, demonstrating a significant reduction in the number of revisions per patient (*p* < 0.01). In the future, comparing this impact on the revision rate with that of fixed-pressure valves is crucial and may better inform valve selection for patients [10].

The predecessor to the *proGAV®2.0* was extensively investigated, with overall survival rates of 81–88.7% [6, 11, 23] in keeping with those reported here. Whilst similar revision rates have been reported in both programmable and fixed-pressure valves [10,13], in this study, both 12-month survival and mean time to revision were significantly greater in the *proGAV®2.0* cohort (Figs. 1 and 2). This supports the wider use of programmable valves, not just in those with CSF over-drainage, but as a safe alternative for hydrocephalus in general.

In the *proGAV®2.0* group alone, de novo and revised valves did not differ significantly in their mean survival (31.9 vs 40.3 months, *p* = 0.10) [21, 22]. The shorter survival seen in de novo valves may be explained by the fact that de novo valves were more frequently placed in a younger cohort in this study (0.9 vs 5 years), a feature that has independently been shown to increase risk of shunt failure [23]. In the *proGAV®2.0* group, patients ≤12 months of age at insertion did have shorter mean valve survival times and cumulative survival at 12 months compared to those >12 months of age (35.3 months and 71.2% vs 37.3 months and 86.3% respectively), though this discrepancy was not significant (*p* = 0.25).

Over-drainage was the indication for revision in 11% of all valve types requiring revision (Table 2). The overall prevalence of over-drainage amongst valves that were replaced by *proGAV®2.0s* was significantly greater than either *proGAV®2.0* or Miethke fixed-pressure valves (*p* < 0.01) (Table 2), though selection bias is likely responsible for this high prevalence. Both *proGAV®2.0s* and fixed-pressure valves had low rates of over-drainage (1.1% vs 1.9% respectively) in this study, though these were relatively small cohorts and further follow-up is required. In those that had a *proGAV®2.0* inserted for over-drainage, no cases recurred following insertion, demonstrating the valve’s efficacy in tackling the condition. Infection as a cause of shunt revision constituted 10% of *proGAV®2.0* revisions but occurred in only 2.2% of all *proGAV®2.0* valves inserted. This is markedly lower than infection rates identified in the literature for other valves [8, 11, 24], as well as other valves in both this series. Other complications rates were similar in prevalence to the literature (6,21,22).

Whilst ICP monitoring may be utilised to determine whether valve revision is necessary prior to surgery, it played a relatively small role in our centre, with only 12.5% having ICP monitoring. Often if clinical suspicion of over-drainage persists despite unremarkable ICP values, surgery may be opted for regardless. As is evidenced by the frequent adjustments made to the valves in this cohort, programmable valves such as the *proGAV®2.0* provide an opportunity to delay the need for surgery, with minimal effort and risk. Anecdotally, practitioners at the centre found the means by which to adjust the *proGAV®2.0*, simple to understand and straightforward to perform.

Limitations to our study include the retrospective nature of the data and the fact that accurately determining aetiology of shunt failure is subject to observer bias. Our study also does not assess the impact of fixed-pressure valves on ventricular metrics or revision rate before and after insertion. This was not examined in the original study either [15]; therefore, we hope to investigate these outcome measures in the near future. The financial cost associated with programmable valves cannot be overlooked, and whilst this study provides encouraging evidence for their wider use, we did not perform a cost-analysis, and this variable should be considered in the future.

## Conclusion

The *proGAV®2.0* programmable valve reduced the need for valve revision in patients with ventricular over-drainage, such that no cases re-occurred during the follow-up period. Both 12-month survival and mean time to revision were significantly greater in *proGAV®2.0* programmable valves compared to Miethke fixed-pressure valves. This study provides evidence to support the wider use of programmable valves*,* but the impact of cost was not assessed, and further investigation is therefore required.

## Data Availability

The datasets generated and/or analysed during the current study are available from the corresponding author on reasonable request.

## References

[1] Aschoff A, Kremer P, Hashemi B, Kunze S (1999) The scientific history of hydrocephalus and its treatment. Neurosurg Rev 22(2-3):67–93 discussion 94-5 10547004 10.1007/s101430050035

[2] Drake JM, Kestle JR, Tuli S (2000) CSF shunts 50 years on—past, present and future. Childs Nerv Syst 16:800–804 11151733 10.1007/s003810000351

[3] Rekate HL (2008) Shunt-related headaches: the slit ventricle syndromes. Childs Nerv Syst 24:423–430 18259760 10.1007/s00381-008-0579-7

[4] Faulhauer K, Schmitz P (1978) Overdrainage phenomena in shunt treated hydrocephalus. Acta Neurochir 45:89–101 742440 10.1007/BF01774384

[5] Pudenz RH, Foltz EL (1991) Hydrocephalus: overdrainage by ventricular shunts. A review and recommendations. Surg Neurol 35:200–212 1996449 10.1016/0090-3019(91)90072-h

[6] Thomale UW, Gebert AF, Haberl H, Schulz M (2013a) Shunt survival rates by using the adjustable differential pressure valve combined with a gravitational unit (proGAV) in pediatric neurosurgery. Childs Nerv Syst 29:425–431 23135777 10.1007/s00381-012-1956-9

[7] Gruber R, Jenny P, Herzog B (1984) Experiences with the anti-siphon device (ASD) in shunt therapy of pediatric hydrocephalus. J Neurosurg 61:156–162 6726390 10.3171/jns.1984.61.1.0156

[8] Freimann FB, Sprung C (2012) Shunting with gravitational valves—can adjustments end the era of revisions for overdrainage-related events? Clinical article. J Neurosurg 117:1197–1204 22998061 10.3171/2012.8.JNS1233

[9] B Braun—Miethke proGAV2.0 online information page. https://www.bbraun.com/en/products/b0/miethke-progav-20.html. Accessed 29 Dec 2020

[10] Agarwal N, Kashkoush A, McDowell MM, Lariviere WR, Ismail N, Friedlander RM (2018) Comparative durability and costs analysis of ventricular shunts. J Neurosurg:1–8. 10.3171/2017.11.JNS172212 10.3171/2017.11.JNS172212 29749912

[11] Rohde V, Haberl EJ, Ludwig H, Thomale UW (2009) First experiences with an adjustable gravitational valve in childhood hydrocephalus. J Neurosurg Pediatr 3:90–93 19278305 10.3171/2008.11.PEDS08154

[12] Beez et al (2014) Role of ventriculoperitoneal shunt valve design in the treatment of pediatric hydrocephalus—a single center study of valve performance in the clinical setting. Childs Nerv Syst. 10.1007/s00381-013-2244-z 10.1007/s00381-013-2244-z 23900632

[13] Riva-Cambrin et al (2016) Risk factors for shunt malfunction in pediatric hydrocephalus: a multicenter prospective cohort study. J Neurosurg Pediatr 17:382–390 26636251 10.3171/2015.6.PEDS14670

[14] Sokratous G, Hadfield O, Van Tonder L, Hennigan D, Ellenbogen J, Pettorini B, Mallucci C (2020) Management of paediatric hydrocephalous with Miethke fixed pressure gravitational valves. The Alder Hey Children’s Hospital experience. Childs Nerv Syst 36(9):2021–2025. 10.1007/s00381-020-04520-x 32020268 10.1007/s00381-020-04520-x

[15] Jamous M, Sood S, Kumar R, Ham S (2003) Frontal and occipital horn width ratio for the evaluation of small and asymmetrical ventricles. Pediatr Neurosurg 39:17–21 12784072 10.1159/000070874

[16] O’Hayon BB, Drake JM, Ossip MG, Tuli S, Clarke M (1998) Frontal and occipital horn ratio: a linear estimate of ventricular size for multiple imaging modalities in pediatric hydrocephalus. Pediatr Neurosurg 29:245–249 9917541 10.1159/000028730

[17] Kulkarni AV, Drake JM, Armstrong DC, Dirks PB (1999) Measurement of ventricular size: reliability of the frontal and occipital horn ratio compared to subjective assessment. Pediatr Neurosurg 31:65–70 10592474 10.1159/000028836

[18] Tervonen J, Ville, Juha L, Jääskeläinen JE, Koponen S, Huttunen TJ (2017) Rate and risk factors for shunt revision in pediatric patients with hydrocephalus—a population-based study. World Neurosurg. 10.1016/j.wneu.2017.02.030 10.1016/j.wneu.2017.02.030 28213196

[19] Simon TD, Whitlock KB, Riva-Cambrin J, Kestle JRW, Rosenfeld M, Dean JM, Holubkov R, Langley M (2012 Jun). Hamblett NM Revision surgeries are associated with significant increased risk of subsequent cerebrospinal fluid shunt infection Paediatr Infect Dis J. 10.1097/INF.0b013e31824da5bd 10.1097/INF.0b013e31824da5bd PMC3356497 22333701

[20] Garton HJ, Kestle JR (2001) Drake JM Predicting shunt failure on the basis of clinical symptoms and signs in children. J Neurosurg 94(2):202–210 11213955 10.3171/jns.2001.94.2.0202

[21] Browd SR, Ragel BT, Gottfried ON, Kestle JR (2006) Failure of cerebrospinal fluid shunts: part I: Obstruction and mechanical failure. Pediatr Neurol 34(2):83–92 16458818 10.1016/j.pediatrneurol.2005.05.020

[22] Browd SR, Gottfried ON, Ragel BT (2006) Kestle JR Failure of cerebrospinal fluid shunts: part II: overdrainage, loculation, and abdominal complications. Pediatr Neurol 34(3):171–176 16504785 10.1016/j.pediatrneurol.2005.05.021

[23] Tuli S, Drake J, Lawless J, Wigg M, Lamberti-Pasculli M (2000) Risk factors for repeated cerebrospinal shunt failures in pediatric patients with hydrocephalus. J Neurosurg 92(1):31–38. 10.3171/jns.2000.92.1.0031 10616079 10.3171/jns.2000.92.1.0031

[24] Sprung C, Schlosser HG, Lemcke J, Meier U, Messing-Jünger M, Trost HA, Weber F, Schul C, Rohde V, Ludwig HC, Höpfner J, Sepehrnia A, Mirzayan MJ, Krauss JK (2010) The adjustable proGAV shunt: a prospective safety and reliability multicenter study. Neurosurgery 66:465–474 20173542 10.1227/01.NEU.0000365272.77634.6B

